# How searcher shoots grow and branch in mature liana mats: the case of *Merremia boisiana*

**DOI:** 10.3389/fpls.2024.1425949

**Published:** 2024-10-04

**Authors:** Qiyu Kuang, Shiying Su, Liang Hu

**Affiliations:** School of Geography and Planning, Sun Yat-sen University, Guangzhou, China

**Keywords:** branching strategies, climbing plants, mortality rate, resprouting rate, stolons, searcher shoots

## Abstract

The proliferation of vigorous lianas usually forms liana canopies over the crowns of host trees and liana mats on the ground of open areas or large forest gaps. While research on liana canopies has increased significantly in recent decades, our understanding of liana mats remains limited. *Merremia boisiana* (Convolvulaceae), a fast-growing liana, forms mature liana mats that can persist for decades, characterized by numerous upright searcher shoots that extend from the liana mats in search of supports. However, the reasons behind the proliferation of these searchers, as well as their growth and branching patterns in mature liana mats that lack support, are not well understood. We hypothesize that searchers are an inevitable phase in the growth rhythm of *M. boisiana* within these mature liana mats. We tested our hypothesis by tracking the lifespan and status of *M. boisiana* searchers during the early, middle, and late periods of the year. Our findings reveal the following: (1) *M. boisiana* searchers exhibit apical dominance and have a short lifespan; over 95% of searchers lost their terminal buds within two months during the early period, while it took only one month during the middle and late periods. (2) The original lateral buds of the searchers also have a limited lifespan, yet the nodes can sprout new lateral buds after the loss of their originals. (3) With the withering of terminal buds, the total number of lateral shoots decreased, while the quantity of long lateral shoots (≥ 50 cm) and their elongation rates increased. (4) Each surviving segment of a searcher typically develops one or two long lateral shoots, none of which grow into searchers. We conclude that the shoots of *M. boisiana* in mature liana mats periodically alternate between searchers and stolons, collectively forming a zigzag branching system. The high rate of lateral bud resprouting may facilitate the rapid recovery of mature liana mats early in the year, while the reduced lifespan of searchers and limited number of long lateral shoots represent effective strategies for *M. boisiana*, balancing the investment and risk associated with foraging in mature liana mats.

## Introduction

Climbing plants and their role in tropical and subtropical forest ecosystems have gained increasing attention in the last four decades (Putz, 1984; Putz and Holbrook, 1991; Schnitzer and Bongers, 2002; Gerwing et al., 2006; Gianoli, 2015; Marshall et al., 2020). Among climbers, stem-twiners are the most widespread and species-rich group, making them a central focus of ecological research (Darwin, 1865; Putz and Holbrook, 1991; Hu et al., 2010). Typically, the stem system of stem-twiners is categorized into climbing “twining stems” and non-climbing “creeping stems” (or stolons) based on their behavior and function (Penalosa, 1984; Gianoli, 2015). Twining stems attempt to find potential supports via elliptical movements of the apex known as circumnutations. Once they encounter a suitably sized support, they climb with a stable spiral structure (Darwin, 1865; Isnard and Silk, 2009; Gianoli, 2015; Hu et al., 2017). Some aggressive liana species produce numerous lateral shoots in the crown of their host plants, forming dense, tangled liana canopies that can eventually lead to the host plants’ death (Penalosa, 1984; Li et al., 2006; Wang et al., 2009; Ladwig and Meiners, 2010). Stem-twiner individuals or ramets can also spread horizontally through the vegetative propagation of stolons, enabling colonization of adjacent clearings. Some exploratory stolons may even establish new populations by spreading through the understory (Penalosa, 1984). In some aggressive species, the intertwined stolons and twining stems can form dense, monospecific liana mats on the ground. While recent research has extensively studied liana canopies (e.g. Marvin et al., 2016; Waite et al., 2022), our understanding of liana mats remains limited.

Liana canopies and mats are frequently observed in areas heavily infested by aggressive stem-twiners such as *Mikania micrantha* (Zhang et al., 2004), *Pueraria montana* var. *lobata* (Kato-Noguchi, 2023) and *Merremia boisiana* (Li et al., 2009; Wang et al., 2009). *M. boisiana* (Convolvulaceae) is a fast-growing, perennial, woody liana native to western Indonesia and Southeast Asia (Staples, 2010; Wang et al., 2005). Over the past three decades, this species has caused devastating damage to local forest ecosystems (Li and Huang, 1996; Wang et al., 2009), yet effective control measures remain elusive. Field observations show that the liana mats of *M. boisiana* can remain stable for decades, characterized by interlaced old stolons in the middle and lower layers and a continuous emergence of new stems in the upper layer throughout the year (Lian et al., 2007). Some of these new stems develop as stolons, while others grow upright as searcher shoots. These searchers extend beyond the liana mats, forage in the air via circumnutation, and eventually collapse if a support was not encountered. Within a liana mat such axes are effectively “doomed” since they are unlikely to find a host plant to climb, given that normally no tree seedlings survive within mature liana mats. This raises the question: why does *M. boisiana* produce so many searchers for what is apparently futile foraging behavior beyond the liana mats?

Generally, lateral branches are thinner than the main branch from which they originate. Li et al. (2006) categorized current-year *M. boisiana* stems into two types—thicker (strong) and thinner (weak)—and reported that the thicker stems were all stolons, while the thinner ones were all twining searchers. This inference was gradually adopted in subsequent studies (Wang et al., 2009; He et al., 2011; Fan et al., 2016). Unfortunately, none of these studies have examined this inference through the lens of the branching system and strategy. As thinner stems, being lighter in weight would likely be advantageous for searchers to expand their foraging range. However, how will the lateral shoots of these searchers, which are even thinner than the searchers themselves, develop? Will all, or at least the vast majority, of these lateral shoots continue to function as new searchers, even if the original searchers fail to find support?

Three observable characteristics in mature *M. boisia*na liana mats challenge the assumption that lateral shoots retain their twining ability: (1) all upright-growing searchers emerge from horizontally growing stolons; (2) all lateral shoots emerge at a steep angle of about 70–80 degrees, making them nearly horizontal; and (3) the middle and lower layers of mature *M. boisiana* liana mats are composed almost entirely of straight branch segments, with intertwined stems being extremely rare. If most of these horizontally growing lateral shoots still possessed the ability to twine, we would expect to find numerous old, twined segments within the liana mats. This discrepancy suggests that the lateral shoots of the upright-growing searchers in mature liana mats may develop into horizontally growing stolons rather than twining stems.

We hypothesize that most of the lateral shoots of the searchers will lose their twining ability and instead grow as stolons, representing an inevitable phase in the branching rhythm of *M. boisiana* within mature liana mats. This branching strategy, involving stolon-like lateral shoots and the early cessation of terminal bud growth, contributes to maintaining the structure of the mat. The surviving segments of searchers and stolons together form the zigzag branching systems within these mats. To test this hypothesis, we tracked the lifespan and development of the main and lateral shoots of *M. boisiana* searchers. We recorded the number and length of lateral shoots on surviving searchers and analyzed their branching strategies. Additionally, we examined the mortality and resprouting rates of lateral buds on *M. boisiana* searchers and discussed their potential impact on liana mat development. Finally, our findings provide new insights into the branching strategies of stem-twiners within liana mats of this aggressive species.

## Methods

**Figure 1:**
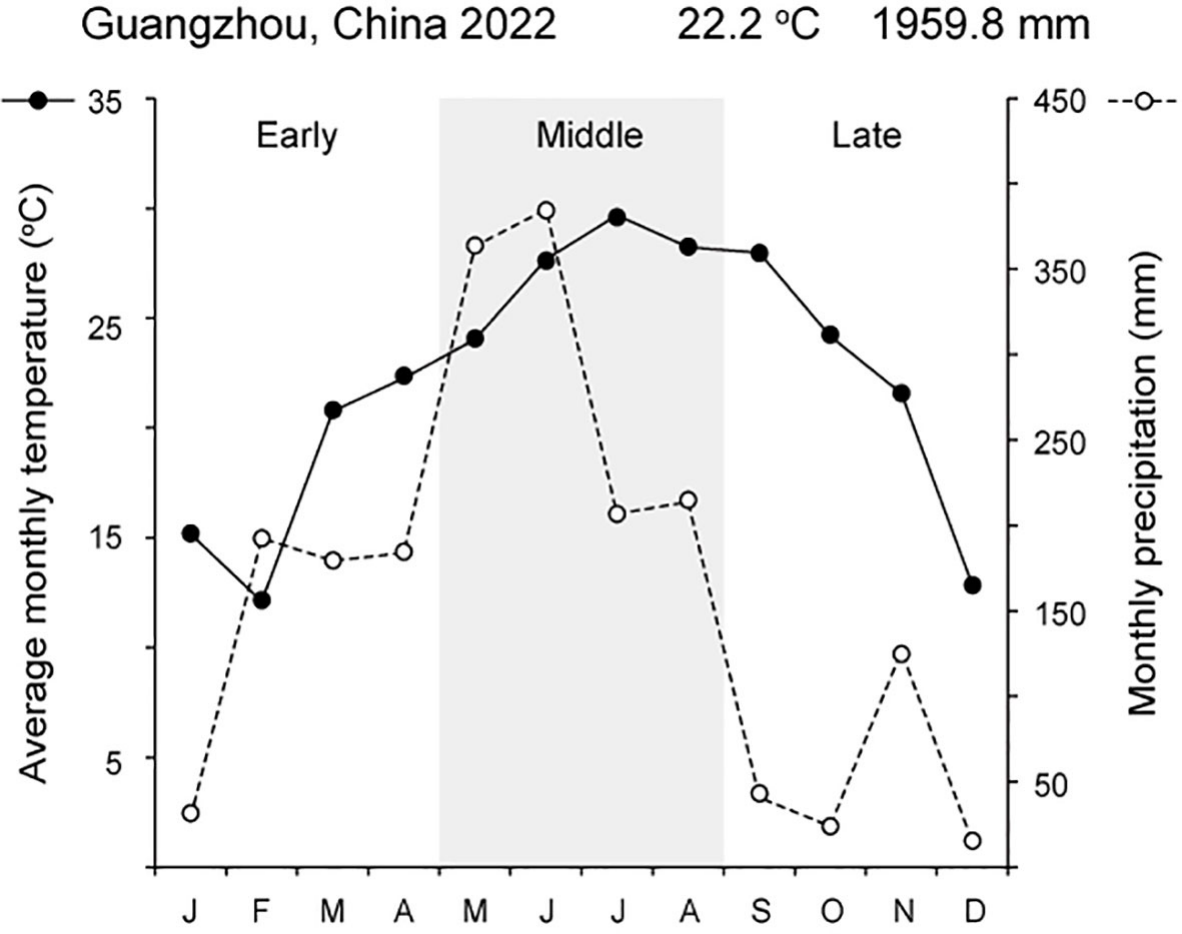
Climate conditions in Guangzhou, China, during 2022, showing the division of the three observation periods.

**Figure 2:**
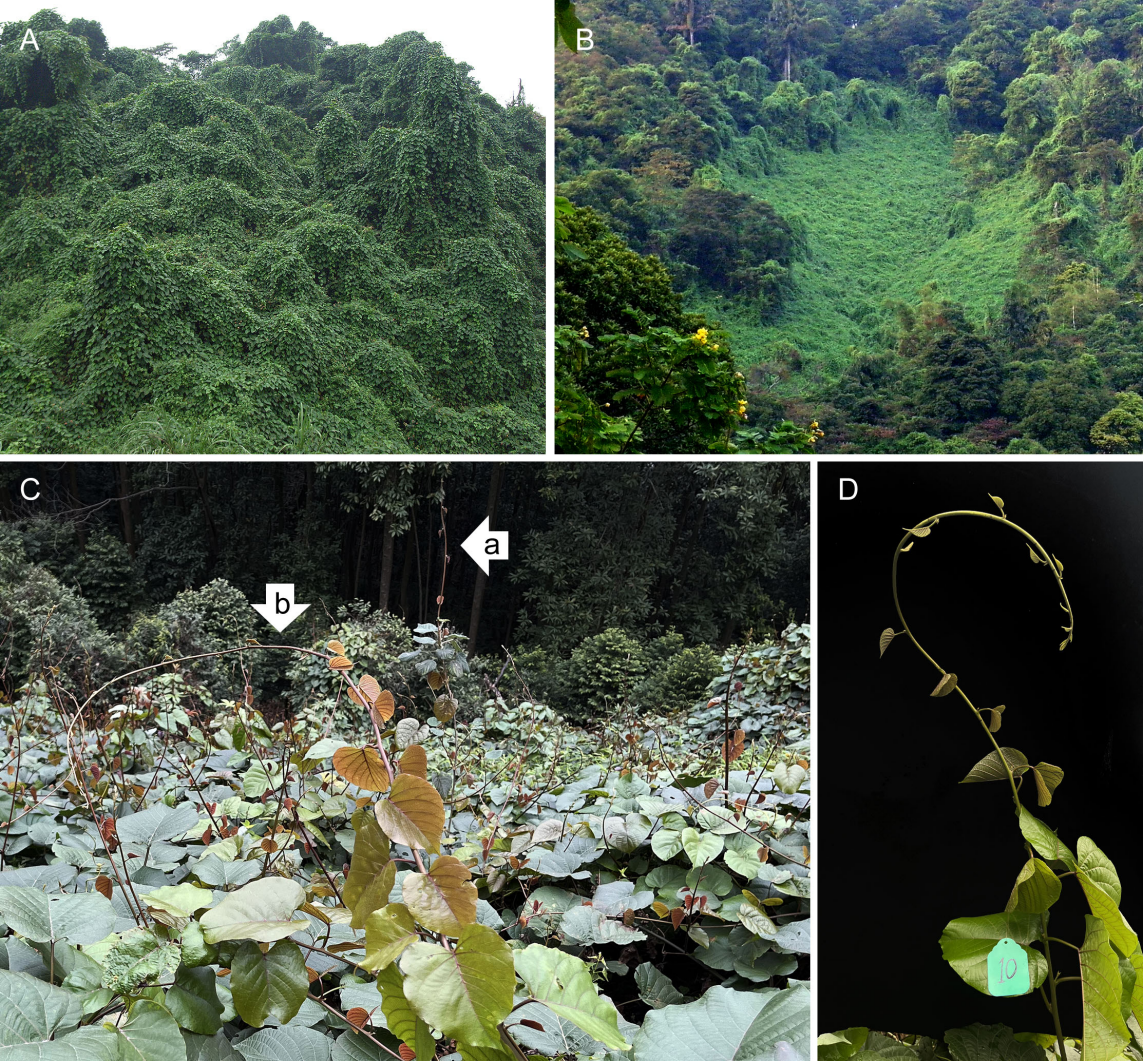
*Merremia boisiana* formations in Guangzhou, China. **(A)** Liana canopy. **(B)** Liana mat. **(C)** A mature liana mat with numerous upright-growing searcher shoots. These searchers may initially grow as self-supporting stems (a), but eventually collapse due to lack of support (b). **(D)** A tagged sample of an *M. boisiana* searcher.

Fieldwork was conducted in 2022 in an area with mature *M. boisiana* liana mats at Longdong Forest Park, northeast of Tianhe District, Guangzhou, China (N 23°13′54″, E 113°21′59″). The study area experiences a subtropical monsoon climate, with an annual temperature of 22.2°C and an annual precipitation of 1959.8 mm in 2022 (**Figure 1**). *M. boisiana* grows year-round in this region (Lian et al., 2007). *M. boisiana* has colonized this area since the 1990s (Xu and Li, 1994), forming a mature liana mat approximately 70–90 cm in height (**Figure 2**) that has persisted for at least two decades. Due to the angle at which most new shoots grow—typically 70–80 degrees—it is challenging to distinguish new searchers from new stolons when they are still very short. Preliminary observations revealed that *M. boisiana* completes internode elongation within about one week, with leaf blade growth following in two weeks. Additionally, it also takes roughly two weeks for buds to sprout from the stolons and extend beyond the liana mats to become searchers. At this stage, the searchers have around ten mature internodes, inactive lateral buds, and are growing vertically as self-supporting stems. This makes it feasible to distinguish them from new stolons, allowing us to begin the random selection of searcher samples for growth dynamic and branching strategy observations.

All field measurements were conducted in 2022, with three observation periods corresponding to early (January to April), middle (May to August), and late (September to December) periods of the year (**Figure 1**). During each period, 25 searchers with intact leaves and lateral buds within the mature liana mats were randomly selected and their growth dynamics and branching strategies were observed at 30-day intervals. Each observation period lasted 90 days, resulting in a total of 75 searchers being observed and measured throughout the study.

**Figure 3:**
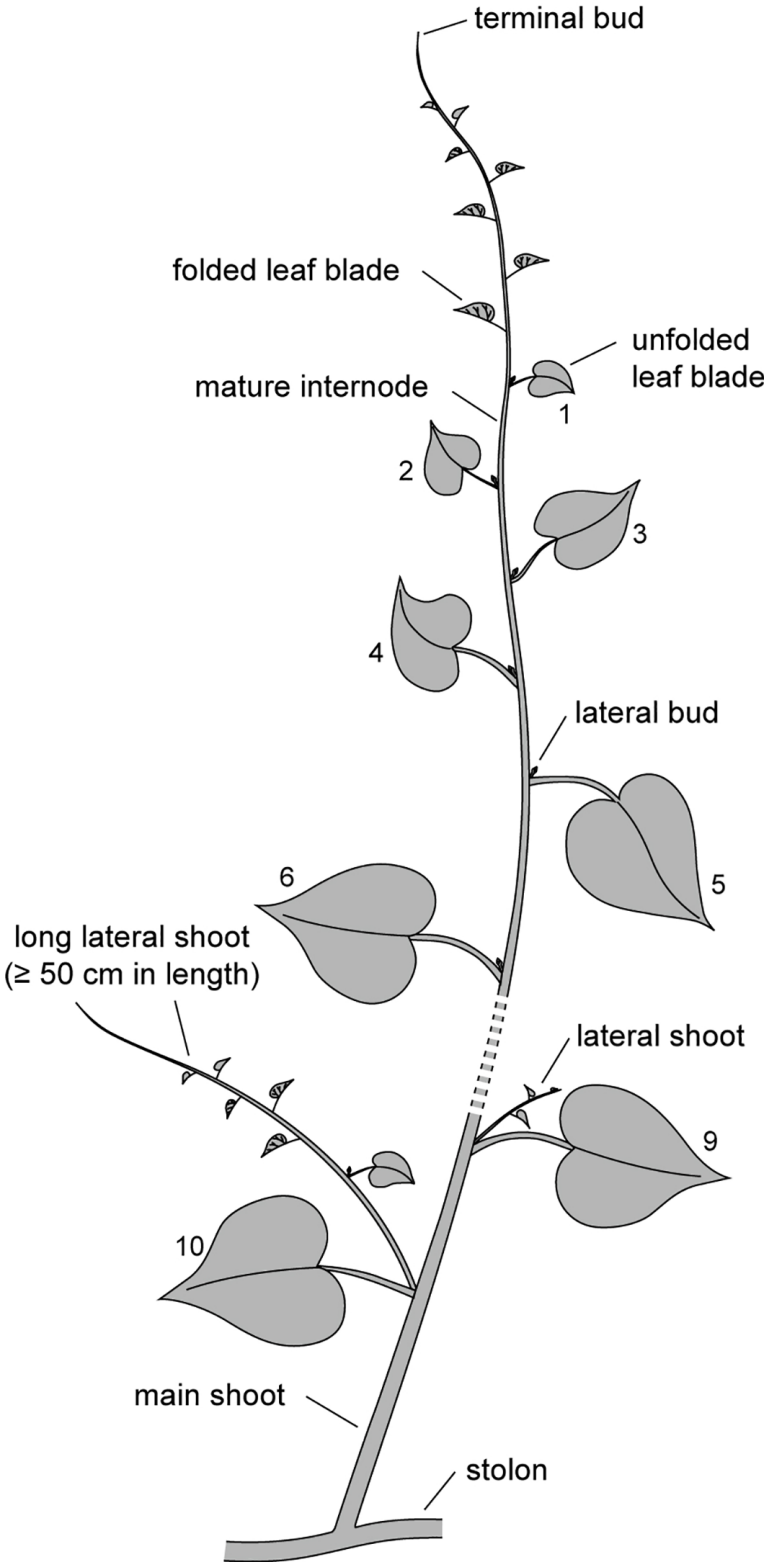
Illustration of a *Merremia boisiana* searcher within mature liana mats.

In the early period, the random selection of searcher samples and the first measurements were taken on January 15. The length of each searcher was precisely measured from the base to the terminal bud using a measuring tape. Since the new leaf blades of the nodes near the terminal tip were still folded (**Figure 3**), the uppermost node with a length of at least 2.0 cm and a fully unfolded leaf blade was designated as the first node. From this point, 10 nodes downward were tagged to monitor the survival status of the leaves and lateral buds (**Figure 3**). Every 30 days, the survival status of searchers, terminal buds, tagged leaves, and lateral buds was recorded, and cumulative mortality rate was calculated. The resprouting rate of lateral buds was determined by calculating the ratio of the number of resprouted lateral buds to the total number of withered lateral buds/shoots. For each searcher, the elongation increments of the main shoot and each lateral shoot were measured. Lateral shoots that reached a length of at least 50 cm were classified as long lateral shoots (*LLS*) (**Figure 3**). The total number of lateral shoots (*N_t_*) and the number of *LLS* (*N_l_*) were counted. Additionally, the length of each lateral shoot (*L*) was measured, with the length of the longest *LLS* represented by *L_m_*. The total length of all lateral shoots (*L_t_*) was calculated for each surviving searcher. At the end of the observation period, the final survival status of the tested searchers and their lateral shoots was evaluated. The procedures for the middle and late-period observations and measurements followed the same protocol as in the early period. The Kruskal-Wallis Test was used to analyze differences in monthly elongation increments (*MEI*) across different groups, while the Mann-Whitney U Test was employed for pairwise comparisons after the Kruskal-Wallis Test identified significant differences.

## Results

Out of the initial sample, six searchers were excluded from the analysis due to damage from artificial defects or unknown causes during the observation periods. The final analysis included a total of 69 searchers, distributed across the early, middle, and late periods, with 22, 23, and 24 stems, respectively (**Table 1**).

**Table 1:** Final survival status of *Merremia boisiana* searchers and their long lateral shoots (*LLS*, ≥ 50 cm in length) during the early, middle, and late periods of the year in Guangzhou, China.

	Early period		Middle period		Late period	
	No.	%	No.	%	No.	%
Tested searchers	22	–	23	–	24	–
Dead/withered searchers	7	31.8	21	91.3	6	25.0
Surviving searchers	15	68.2	2	8.7	18	75.0
without *LLS*	1	4.5	1	4.3	9	37.5
with 1 *LLS*	4	18.2	1	4.3	9	37.5
with 2 *LLS*	8	36.4	0	–	0	–
with 3 *LLS*	2	9.1	0	–	0	–

Each observation period lasted three months.

### Mortality rate of searchers, lateral shoots, buds, and leaves

#### Searchers and lateral shoots

A key characteristic of *M. boisiana* searchers is their short lifespan, particularly evident in the middle period. Overall, approximately half of the searchers (35 out of 69 stems) survived three months. Among these, two-thirds (24 out of 35 stems) developed at least one long lateral shoot (*LLS*) by the end of the period (**Table 1**). During the early period, 68.2% of the searchers survived three months, and the vast majority of these (93.3%) had at least one *LLS* (**Table 1**). However, survival dropped sharply in the middle period, with only two searchers (8.7%) surviving three months, and just one of these had an *LLS*. During the late period, 75% of the searchers survived three months, with half of which producing an *LLS* (**Table 1**).

#### Buds and leaves

The terminal and lateral buds of *M. boisiana* searchers also exhibited a short lifespan, though their survival patterns varied. In the early period, over half of the terminal buds survived the first month, but nearly all withered by the end of the second month (**Table 2**). In the middle and late periods, survival rates were even lower; only 4.5% and 4.2% of terminal buds survived the first month, respectively, with no terminal buds surviving beyond the second month (**Table 2**).

**Table 2:** Cumulative mortality rate (%) of *Merremia boisiana* searcher shoots and their terminal buds, original lateral buds, and leaves during the early, middle, and late periods of the year in Guangzhou, China.

Mortality	Early period			Middle period			Late period		
	I	II	III	I	II	III	I	II	III
Searcher shoots	0.0	4.5	31.8	13.0	43.5	91.3	0.0	12.5	25.0
Terminal buds	45.5	95.5	100	95.5	100	-	95.8	100	-
Original lateral buds	1.6	27.3	83.3	85.3	98.2	100	27.1	78.4	89.8
Leaves	3.2	15.3	96.8	51.6	100	-	6.7	76.9	88.2

Each observation period lasted three months, with I, II, and III representing the first, second, and third months, respectively.

In contrast, the original lateral buds generally outlasted the terminal buds. During the early period, most original lateral buds survived at least two months, with a final survival rate of 16.7%. In the middle period, although the vast majority of original lateral buds were lost in the first month, their cumulative mortality rate was about 10% lower than that of terminal buds (**Table 2**). By the end of the middle period, no original lateral buds remained. During the late period, over 70% of the original lateral buds survived the first month, about 20% lived on through the second month, and only about 10% survived the full three months (**Table 2**).

The cumulative mortality rates and peak mortality of the leaves were similar to those of the lateral buds (**Table 2**). Across all three periods, the cumulative mortality rate of leaves was lower than that of lateral buds during the first month, but higher at the end of the observation period (**Table 2**).

### Resprouting of Lateral Buds

**Figure 4:**
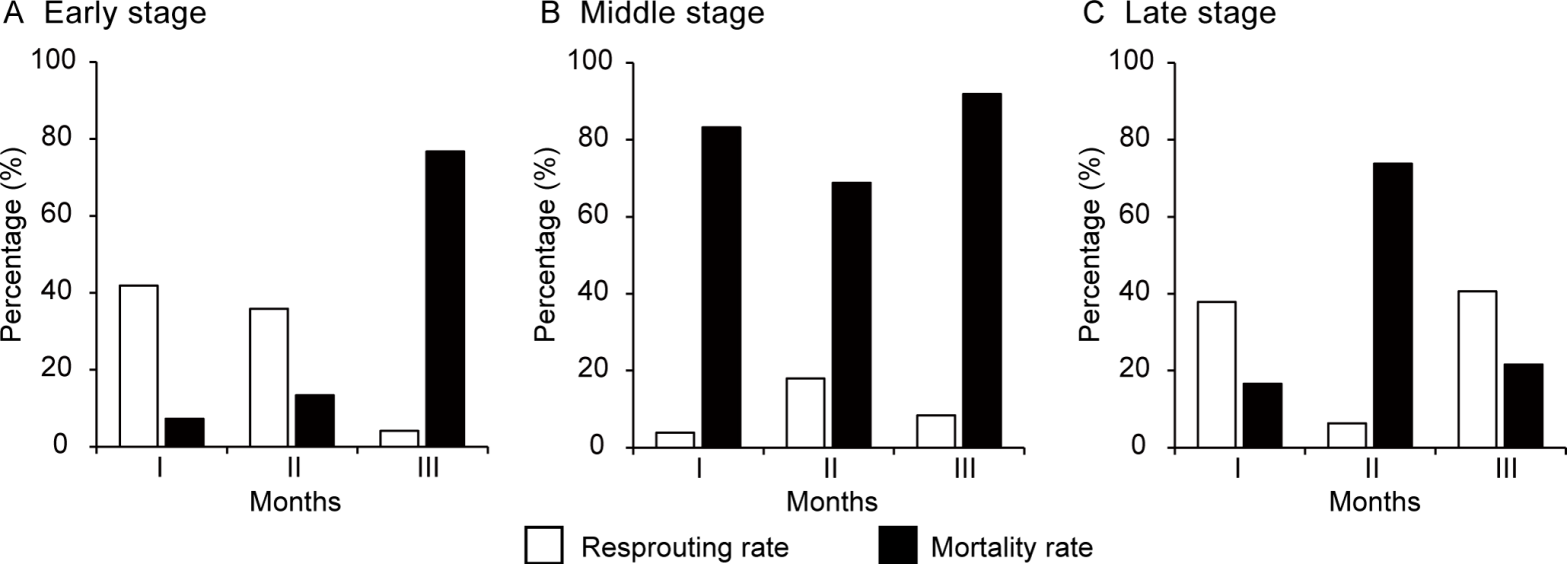
Monthly resprouting and mortality rates of the lateral buds on *Merremia boisiana* searchers during the early, middle, and late periods of the year in Guangzhou, China. Each observation period lasted three months, with I, II, and III representing the first, second, and third months, respectively.

After the loss of an original lateral bud, *M. boisiana* nodes were capable of forming new lateral buds. Generally, a high resprouting rate of lateral buds was associated with a decrease in their overall mortality rate (**Figure 4**). The highest monthly resprouting rates were observed at the beginning of the early period (41.7%) and at the end of the late period (40.0%) (**Figures 4A, C**). Conversely, during the middle period, resprouting was minimal, with monthly rates of only 3.2%, 17.5%, and 7.7% across the three months (**Figure 4B**).

### Growth of main and lateral shoots

#### Main shoots

As long as the terminal buds of *M. boisiana* searchers remained intact and active, the main shoots exhibited a dominant elongation rate.

**Figure 5:**
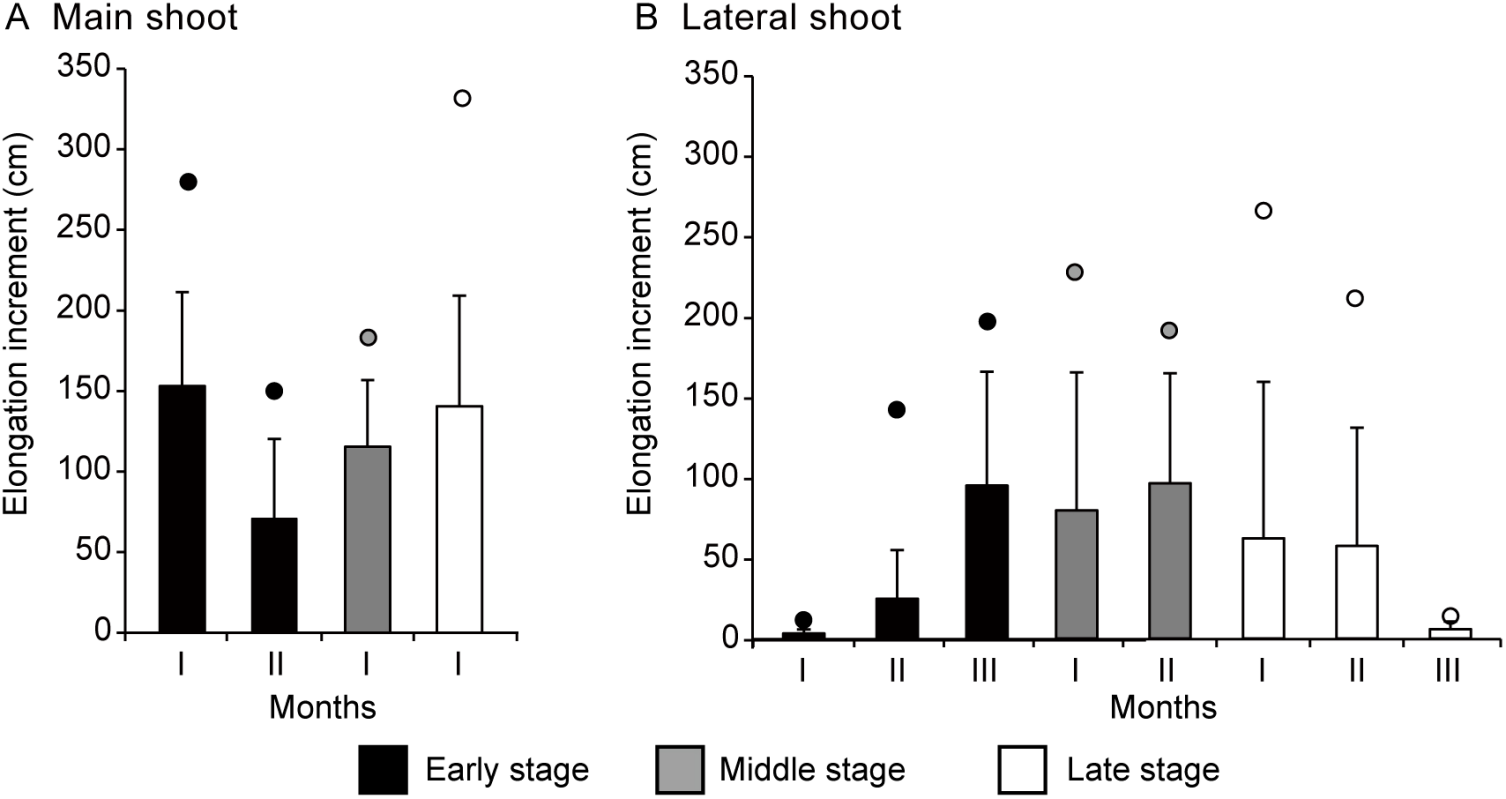
Monthly elongation growth of **(A)** main and **(B)** lateral shoots of *Merremia boisiana* searchers during the early, middle, and late periods of the year in Guangzhou, China. Each observation period lasted three months, with I, II, and III representing the first, second, and third months, respectively. No data are available for the main shoots during the second month of the middle and late periods, as all main shoots lost their terminal buds within the second month. Bars indicate average values, while dots indicate maximum values.

Despite the loss of the terminal apex in many main shoots within the first month, the Kruskal-Wallis Test produced a p-value of less than 0.05 across all three periods, suggesting a significant difference in the mean monthly elongation increments (*MEI*) between the groups (periods and shoots). Specifically, a highly significant difference in *MEI* between the main and lateral shoot groups was observed in both the early (p < 0.001) and late (p < 0.001) periods, but not in the middle period (p = 0.320). Additionally, there was no significant difference in the *MEI* of the first month when comparing any two of the three periods (p > 0.1). During the first month, the *MEI* of the main shoots reached 153.0 cm, 115.3 cm, and 140.4 cm in the early, middle, and late periods, respectively, with maximum values of 280.0 cm, 183.1 cm, and 331.9 cm (**Figure 5A**). In the early period, although the *MEI* of the remaining main shoots decreased to 69.9 cm in the second month, it still surpassed the growth of lateral shoots during the same period (**Figures 5A, B**). No data are available for the main shoots during the second month of the middle and late periods, as all main shoots lost their terminal buds within the second month.

#### Lateral shoots

**Figure 6:**
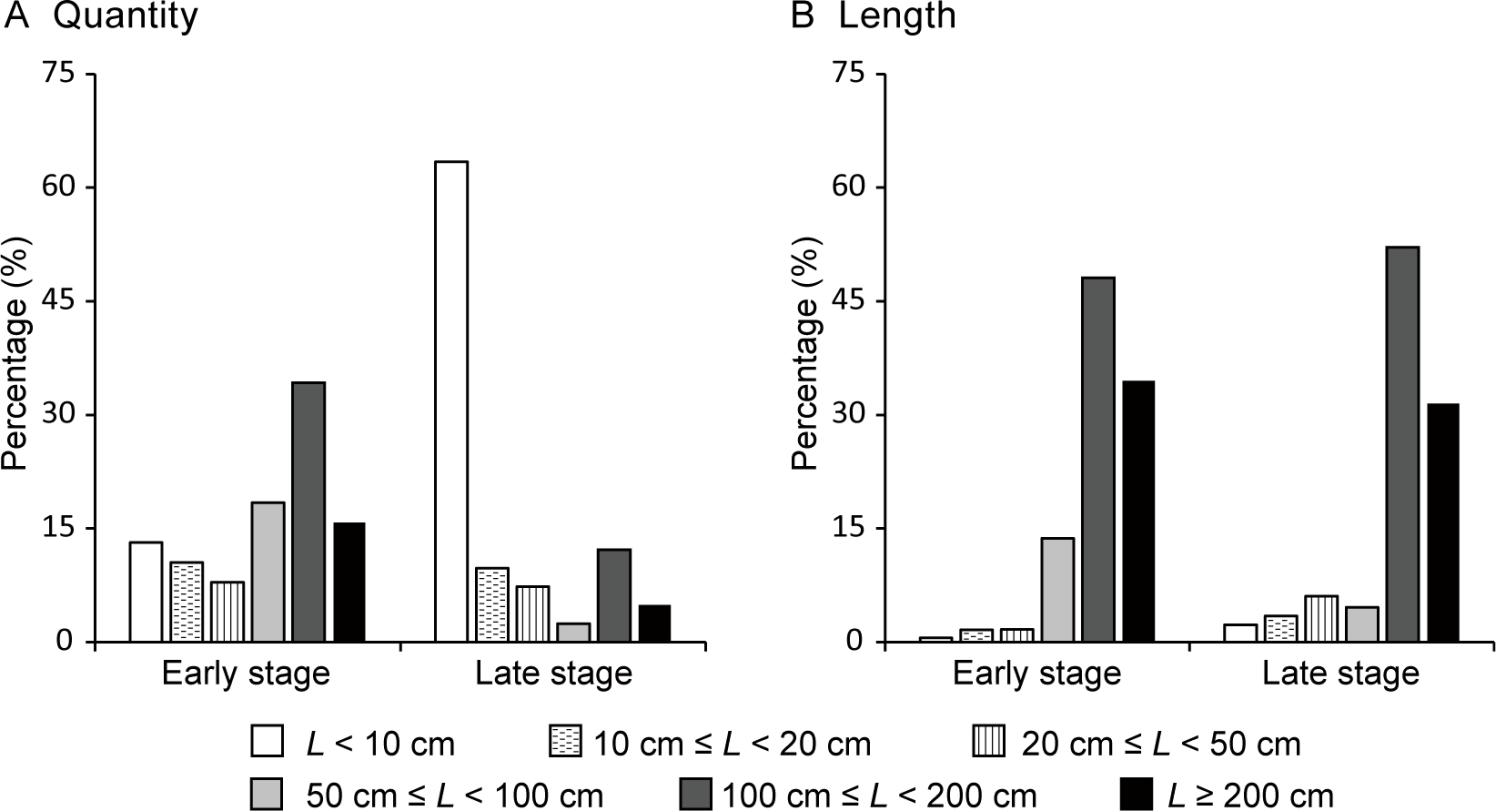
Frequency distributions of the **(A)** number and **(B)** length (*L*) of surviving lateral shoots on *Merremia boisiana* searchers at the end of the early and late periods of the year in Guangzhou, China.

The elongation of lateral shoots was primarily driven by a small number of vigorous *LLS*. Overall, only 8.7% of the surviving lateral buds developed into *LLS* by the end of the first month. At the end of the early period, only 38 lateral shoots remained, of which 26 (68.4%) were *LLS*, accounting for 96.3% of the total lateral shoot length (*L_t_*) (**Figure 6**). The 19 longest *LLS*, each at least 100 cm long, made up only half of the total number of lateral shoots (*N_t_*) but contributed to 82.6% of the *L_t_* (**Figure 6**). Similarly, by the end of the late period, 41 lateral shoots remained, with eight (19.5%) being *LLS*, representing 88.2% of the *L_t_*. The seven longest *LLS*, each at least 100 cm long, accounted for 17.1% of the *N_t_* but 83.6% of the *L_t_*. In contrast, the shortest surviving lateral shoots (less than 10 cm in length) comprised 63.4% of the *N_t_* but only 2.3% of the *L_t_*.

As the terminal buds of *M. boisiana* searchers withered, the elongation rate of lateral shoots increased. In the early period, the *MEI* of lateral shoots rose from 3.1 cm in the first month to 24.6 cm in the second month and 94.5 cm in the third month (**Figure 5B**). In the middle and late periods, when the lifespan of the main shoots was significantly shorter, the *MEI* of lateral shoots reached 79.1 and 62.0 cm in the first month, respectively. However, by the end of the year, when the weather cooled, the average *MEI* of lateral shoots dropped to 5.8 cm (**Figure 5B**).

Throughout the observation period, *N_t_* decreased, while *L_t_* and *N_l_* increased. By the end of the early period, *N_l_* had increased to 1.9, and the length of the longest *LLS* (*L_m_*) had increased to 176.1 cm (**Table 3**). Only one of the longest *LLS* lost its shoot apex during the first observation period (**Table 3**). In the late period, however, a similar pattern emerged as with the terminal shoot of searchers: two-thirds of the longest *LLS* lost their shoot apex within the third month, leading to a significant decrease in *L_m_* (**Table 3**).

**Table 3:** Growth of lateral shoots on *Merremia boisiana* searchers that survived three months with at least one long lateral shoot (*LLS*, ≥ 50 cm in length).

Period	Stem no.	First month				Second month				Third month			
		*N_t_*	*N_l_*	*L_t_*	*L_m_*	*N_t_*	*N_l_*	*L_t_*	*L_m_*	*N_t_*	*N_l_*	*L_t_*	*L_m_*
Early	1	9	0	13.3	–	9	0	76.5	–	2	1	211.4	187.4
	2	8	0	34.3	–	8	4	400.4	138.6	5	3	550.1	233.5*
	3	10	0	7.8	–	9	0	41.0	–	2	1	118.4	99.4
	4	6	0	6.2	–	4	0	28.4	–	2	2	167.8	94.6
	5	8	0	20.1	–	7	2	188.7	58.5	1	1	95.2	95.2
	6	9	0	17.8	–	9	3	245.9	76.7	3	2	445.6	270.8
	7	9	0	9.3	–	8	2	179.4	64.0	2	2	365.8	247.9
	8	9	0	12.5	–	8	1	117.9	94.6	4	2	477.5	262.6
	9	10	0	10.0	–	9	0	72.2	–	2	2	293.7	208.1
	10	10	0	57.5	–	9	2	357.8	154.7	5	2	324.7	150.1
	11	9	0	14.2	–	8	1	117.9	60.5	1	1	110.1	110.1*
	12	10	0	14.4	–	9	1	149.6	92.0	2	2	238.1	169.4*
	13	9	0	16.9	–	9	1	108.0	55.3	2	2	172.7	116.3
	14	8	0	5.5	–	8	0	124.8	–	3	3	585.4	219.5
	*Mean*	*8.9*	*0*	*17.1*		*8.1*	*1.2*	*157.8*	*88.3*	*2.6*	*1.9*	*296.9*	*176.1*
Middle	1	5	2	348.2	228.1	0	0	–	–	1	1	60.6	60.6*
Late	1	6	1	274.3	269.0	1	1	230.0	230.0	2	1	171	169.2*
	2	10	0	15.7	–	4	1	197.7	196.5	4	1	196.8	195.6
	3	8	1	247.3	239.2	1	1	273.5	273.5	1	1	273.3	273.3
	4	9	0	3.1	–	4	1	173.4	172.1	3	1	169.7	168.6
	5	10	0	6.8	–	6	1	233.5	211.9	6	1	247.9	214.7
	6	10	1	172.2	166.8	4	0	1.7	–	3	1	139.1	138.2*
	7	9	1	176.7	171.3	3	0	49.4	–	3	1	51.2	50.0*
	8	9	1	64.3	60.1	6	1	141.0	138.9	5	1	146.2	136.0
	9	8	0	5.0	–	4	1	115.2	102.8	3	1	81.6	71.2*
	*Mean*	*8.8*	*0.6*	*107.3*	*181.3*	*3.7*	*0.8*	*157.3*	*189.4*	*3.3*	*1.0*	*164.1*	*157.4*

Observations were conducted during the early, middle, and late periods of the year in Guangzhou, China. Each observation period lasted three months. *N_t_*: total number of lateral shoots. *N_l_*: number of *LLS*. *L_t_*: total length of lateral shoots. *L_m_*: length of the longest *LLS*. In the far-right column, the longest *LLS* that lost the apex of the lateral shoot is underlined, and an asterisk indicates that the longest *LLS* changed or was been replaced during the three-month observation period.

As expected, all surviving *LLS* developed as stolons, with none growing as searchers. However, not all of the longest *LLS* maintained their apical dominance throughout growth, indicating that they were also at risk of withering and being replaced. For example, the only searcher that survived the full three months with *LLS* during the middle period sprouted five lateral shoots in the first month, two of which became *LLS*, with the longest reaching 228.1 cm long. In the second month, all lateral shoots died. However, in the third month, one lateral bud resprouted and grew to 60.6 cm in length (**Table 3**). Similar patterns were observed in the early and late periods (**Table 3**). Overall, the longest *LLS* changed in one-third of the searchers that survived the three-month period.

## Discussion

*M. boisiana* searchers that emerge at forest edges are often characterized by rapid growth and are multi-branched, particularly in environments where the density of suitable supports is high. However, in mature liana mats, the vertical and climbing growth of searchers is significantly suppressed due to the lack of available supports. As a result, the branching strategy of searchers within these mats shifts from being multi-branched to less-branched, with all surviving lateral shoots developing exclusively as new stolons.

### Branching strategies in liana mats

The branching strategy of vines may vary significantly across species. Invasive and aggressive stem-twiners often produce more branches than native vines (Zhang et al., 2011). For a given species, factors such as the availability of supports, stem types, and behavioral phases also influence branching strategies. In some species, unsupported plants produce more branches than those with support (den Dubbelden and Oosterbeek, 1995). However, in other species, the opposite is true (Gartner, 1991). The twining stems of *Ipomoea phillomega* branch from almost every node, while its stolons do not branch as long as the apical bud is intact (Penalosa, 1984). Additionally, a twining stem that has just encountered a support of suitable size may initially prioritize upward growth, producing fewer lateral shoots, but once securely attached to the supports, it may produce many lateral shoots.

At forest edges, supported searchers of *M. boisiana* remain active for longer periods and produce numerous branches, most of which are also searchers. However, our results confirmed that the majority of terminal buds on *M. boisiana* searchers in mature liana mats are short-lived. Although many lateral buds may become active, most die quickly, leaving only one or two *LLS* that grow as stolons. The production of these “doomed” searchers in mature liana mats seems to be an inefficient use of energy. The shortened lifespan of searchers and limited number of *LLS* therefore represent a cost efficiency strategy for *M. boisiana*, balancing investments and risks associated with foraging behavior during growth in mats.

**Figure 7:**
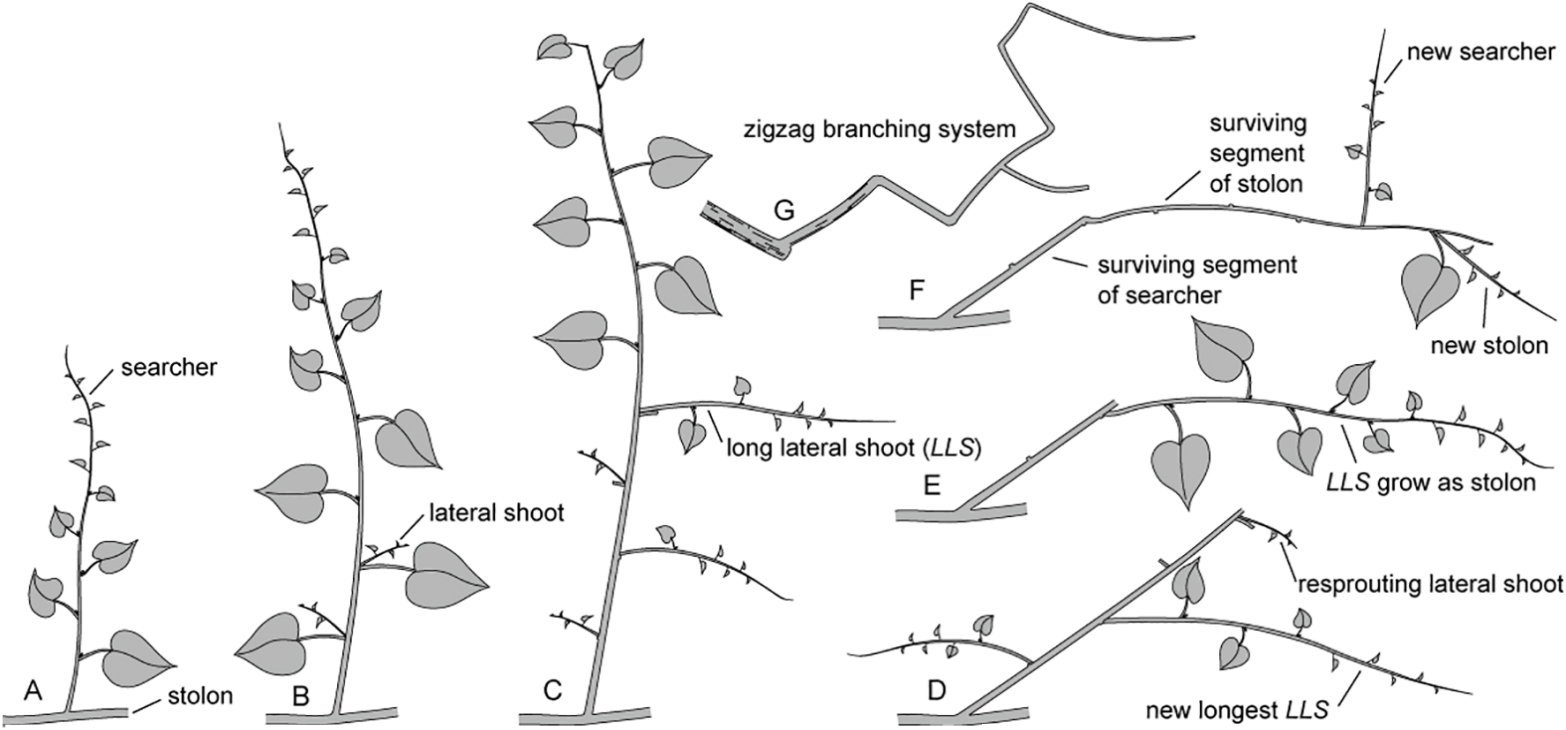
Growth dynamics of *Merremia boisiana* searchers and stolons within mature liana mats. **(A)** A new searcher sprouts from a stolon. **(B)** Lateral shoot growth remains suppressed as long as the terminal bud is intact and active. **(C)** Lateral shoot growth increases when the searcher loses its terminal bud tip. **(D)** Upon withering of the main shoot, the surviving segment of the searcher falls over. The original longest long lateral shoot (*LLS*) in the previous diagram has wilted and been replaced by a resprouting lateral shoot from the same node. **(E)** Most lateral shoots have withered, with the surviving *LLS* now growing horizontally as a new stolon. **(F)** As the new stolon loses its tip, secondary searcher and/or stolon shoots may sprout from the surviving segment. **(G)** The zigzag branching system consists of stolons and surviving searcher segments at all levels.

According to Li et al. (2006), the terminal buds of *M. boisiana* stolons live about two weeks longer than those of searchers in liana mats. Considering the findings that in the upper layer of mature liana mats, all searchers grow from stolons and all surviving *LLS* of the searchers grow as stolons, it is reasonable to conclude that the zigzag branching systems of *M. boisiana* in mature liana mats are primarily composed of segments of both stolons and searchers (**Figure 7**).

The branching strategy of *M. boisiana* searchers in mature liana mats also vary throughout the year. Like other aggressive lianas (Sakai et al., 2002), *M. boisiana* produces many new searchers in the liana mat early in the year. The survival rate of these early searchers is the highest of the year, with most surviving searchers producing at least two *LLS* that grow as stolons. According to He et al. (2011), all stolons of *M. boisiana* that survive the early periods are likely to persist until the end of the year. Combined with the highest resprouting rate of lateral buds, these advantages ensure that *M. boisiana* ramets can recover early in the year and occupy available habitat quickly.

In the middle period of the year, the mortality rate of searchers reaches its peak, while the resprouting rate of lateral buds is at its lowest. Several factors contribute to this high mortality. First, the lack of support plays a significant role. For example, the mortality rate of *Wisteria floribunda* ramets that held onto host trees (56.5%) is significantly lower than that of unsupported ramets (84.6%) (Sakai et al., 2002). Second, the high mortality rate of searchers in the middle period may also be linked to the rapid elongation of the shoots. Overall, *M. boisiana* shoots elongate more rapidly under higher water and heat conditions compared to lower water and heat conditions (Huang et al., 2015; Fan et al., 2016). During the middle period, water and heat conditions were at their peak for the year (**Figure 1**). The rapid elongation of searchers may cause them to fall over and wither prematurely, further increasing the mortality rate.

By the end of the year, the searchers appear to recover somewhat from the high mortality experienced in the previous period. Although the average number of *LLS* is now only half of what it was earlier in the year, the average number of surviving lateral shoots is the highest observed all year. While most of these shoots are very short, they may develop into stolons at the beginning of the following year, thereby resuming the periodic cycle of searcher and stolon growth.

The primary benefit of producing dense searchers appears to be the prevention of tree seedlings from growing out of the liana mat, though nearly none would survive anyway due to the shading by the thick liana mats (Lian et al., 2007). Another possible explanation for the abundance of searchers in mature *M. boisiana* liana mats is that the shoots there follow the same growth strategy as those at forest edges, periodically alternating between searchers and stolons (Li et al., 2006). First, the distinction between searchers and stolons is a rough categorization based on the current behavior of the shoots. If a searcher fails to find support, it may fall to the ground, forage again, or transform into a stolon (Hegarty, 1990). Conversely, a stolon may become a searcher if it encounters a support of suitably size (Penalosa, 1984). Second, there appears to be a limit to the duration of searchers’ foraging behavior. *Humulus lupulus*, for example, stops circumnutating after five days of failing to find support (Darwin, 1865). Gianoli (2015) suggested that these climbers may have a “give-up” time concerning support finding. In the case of *M. boisiana* searchers, this give-up time likely coincides with the withering of terminal buds, which may explain why all the *LLS* of surviving searchers were stolons.

It is therefore reasonable to assume that stolons also have a critical point, or kind of “give-up” time. Our results showed that most of the longest surviving *LLS* lost their shoot apex by the end of the year. Undoubtedly, new searchers will sprout from the lateral buds of these surviving stolon segments at the beginning of the next year (**Figure 7**). Additionally, Li et al. (2006) reported that *M. boisiana* stolons initially grow in a straight line. As they extend, the terminal buds of stolons may begin foraging and searching. It appears that *M. boisiana* stolons have two strategies for forming new searchers: they can either transform into searchers themselves or to sprout new searchers from their lateral buds.

### Liana mats stability and balance

Soffiatti et al. (2022) recently described four possible phases of classic twining stems in forest canopies: (1) a self-supporting stem that has not yet encountered a support, (2) a pendulous stem that has not encountered a support and is no longer self-supporting, (3) a climbing stem that has encountered a support and developed twining on it, and (4) a fixed stem that has developed a completely fixed segment around a support. The twining searchers of *M. boisiana* in mature liana mats differ from these classic twining stems in that the last two phases are typically unattainable due to the scarcity of available supports. Darwin (1865) observed that, in some cases, the flexible shoots of a single species may twine together, forming a cable-like structure that provides mutual support. Gianoli (2015) referred to this phenomenon as “self-twining,” noting that it often occurs when vines grow beyond the height of a short support or fail to find a suitable support.

Our field observations show that self-twining is common in areas occupied by other stem-twiners, such as *M. micrantha*, but is notably absent in the mature liana mats of *M. boisiana*. The terminal buds of *M. boisiana* searchers are short-lived compared to stolons (Li et al., 2006), suggesting that searchers play a lesser role in the formation of branching systems within mature liana mats. Although *M. boisiana* searchers may occasionally intertwine, self-twining does not appear to extend their lifespan. This is evidenced by the fact that the vast majority of the middle and lower layers of the mature *M. boisiana* liana mats consist of straight stems rather than intertwined ones. This feature is a key distinction between a liana mat and a liana trellis, the latter being a net-like structure formed by “trellis-forming stems” (i.e. twining stems) that are intertwined in the air (Soffiatti et al., 2022).

It is intriguing that *M. boisiana* liana mats can remain stable for decades without increasing in thickness. Similar stability has also been observed in other stem-twiners, such as *Schisandra repanda* and *Celastrus orbiculatus* (Ichihashi and Tateno, 2011). Additionally, while *M. boisiana* flowers are abundant in liana canopies, the stems rarely flower within mature liana mats (personal observation). Even when flowering does occur, most of the flowers fall off prematurely and rarely develop into fruits. The underlying mechanism for this remains unclear. Although lianas may have a genetically programmed strategy to produce searchers specifically to find support, we speculate that this behavior may also be driven by an instinctual urge for sexual reproduction, as they need to reach the canopy, or at least grow sufficiently high, to flower.

The total availability of light, water, and soil nutrient remains relatively stable in mature liana mats, making it seemingly risky to invest substantial resources into growing numerous searchers. The energy consumed in this “doomed” foraging behavior could potentially reduce the energy available for sexual reproduction. However, as noted by Lian et al. (2007), the biomass of *M. boisiana* in mature liana mats is primarily concentrated in old stolons with current-year stems comprising only about one-fifth of the total biomass. Assuming that the current year’s searchers and stolons contribute equally to the biomass, this means that the current year’s searchers only account for about 10% of the total biomass. If *M. boisiana* can reproduce sexually near the ground under nutrient-sufficient conditions, is the 10% biomass loss the key reason for the almost complete lack of flowering in mature vine mats? Or is the absence of flowering near the ground an inherent life strategy of this species? We believe further research is needed to determine this, such as providing artificial supports in mature liana mats to test whether searchers that climb to the top will flower.

The actual biomass allocated to the surviving segments of searchers consist of only a few internodes in the lower part of the shoot (**Figure 7**). Additionally, it seems that very few of these one-year-old stems can survive for long periods; otherwise, the thickness of the liana mats would continue to increase.

## Conclusion

The branching patterns of searcher shoots and stolons are crucial for understanding growth cycle and dynamics in liana canopies and mats formed by stem-twiners. While previous studies have primarily focused on forest edges or liana canopies, where interactions between lianas and trees are prevalent, this study provides new insights into the growth and branching patterns of *M. boisiana* searcher shoots within mature liana mats, where host trees are scarce. Our findings reveal that the growth of *M. boisiana* searchers in these mats exhibit apical dominance and is characterized by a shortened lifespan, a high resprouting rate of lateral buds, and a few but vigorous *LLS*. Importantly, we confirmed that the surviving *LLS* are all stolons, demonstrating that *M. boisiana* shoots in these environments periodically alternate between searchers and stolons. This alternation results in a zigzag branching system within the liana mats, composed of both stolon and surviving searcher segments. We suggest that the ecological significance of liana mats and the role of searchers within them should be given greater attention in future studies.

## Data Availability

The original contributions presented in the study are included in the article/supplementary material. Further inquiries can be directed to the corresponding author/s.
